# B‐cell lymphoma‐2 family proteins‐activated proteases as potential therapeutic targets for influenza A virus and severe acute respiratory syndrome coronavirus‐2: Killing two birds with one stone?

**DOI:** 10.1002/rmv.2411

**Published:** 2022-11-30

**Authors:** Sourabh Soni, Yohannes A. Mebratu

**Affiliations:** ^1^ Division of Pulmonary, Critical Care, and Sleep Medicine Department of Internal Medicine The Ohio State University Wexner Medical Center Columbus Ohio USA

**Keywords:** Bcl‐2, Influenza A Virus, SARS‐CoV‐2

## Abstract

The COVID‐19 pandemic caused by severe acute respiratory syndrome coronavirus‐2 (SARS‐CoV‐2) has led to a global health emergency. There are many similarities between SARS‐CoV‐2 and influenza A virus (IAV); both are single‐stranded RNA viruses infecting airway epithelial cells and have similar modes of replication and transmission. Like IAVs, SARS‐CoV‐2 infections poses serious challenges due to the lack of effective therapeutic interventions, frequent appearances of new strains of the virus, and development of drug resistance. New approaches to control these infectious agents may stem from cellular factors or pathways that directly or indirectly interact with viral proteins to enhance or inhibit virus replication. One of the emerging concepts is that host cellular factors and pathways are required for maintaining viral genome integrity, which is essential for viral replication. Although IAVs have been studied for several years and many cellular proteins involved in their replication and pathogenesis have been identified, very little is known about how SARS‐CoV‐2 hijacks host cellular proteins to promote their replication. IAV induces apoptotic cell death, mediated by the B‐cell lymphoma‐2 (Bcl‐2) family proteins in infected epithelia, and the pro‐apoptotic members of this family promotes viral replication by activating host cell proteases. This review compares the life cycle and mode of replication of IAV and SARS‐CoV‐2 and examines the potential roles of host cellular proteins, belonging to the Bcl‐2 family, in SARS‐CoV‐2 replication to provide future research directions.

**Abbreviations**
ACE2: angiotensin‐converting enzyme 2
AEC2(s): alveolar epithelial cell(s)
Apaf‐1: apoptotic protease activating factor‐1
API5: apoptotic inhibitor 5
Bcl‐2: B‐cell lymphoma‐2
BH: bcl‐2 homology
bik^−/−^
: *bik* knockout
bik^+/+^
: *bik* wild‐type
Bok: bcl‐2 ovarian killer
CNS: central nervous system
CoV(s): coronavirus(es)
COVID‐19: coronavirus disease 2019
cRNA: complementary RNA
DIABLO: direct inhibitor of apoptosis‐binding protein with low pI (isoelectric point)
E: envelope
ER: endoplasmic reticulum
HA: haemagglutinin
HAEC(s): human airway epithelial cell(s)
HCoV: human coronaviruses
Hpi: hours post infection
IAV: influenza A virus
M: matrix
MAECs: mouse airway epithelial cells
MAPK: mitogen‐activated protein kinase
Memb: membrane
MERS: middle east respiratory syndrome
MIS: mitochondrial intermembrane space
MOMP: mitochondrial outer membrane permeabilization
N: nucleocapsid
NA: neuraminidase
NLS(s): nuclear localisation signals
NP: nucleoprotein
NS1: non‐structural protein 1
Nsp: nonstructural proteins
OMM: Outer mitochondrial membrane
ORF(s): open reading frames
PA: polymerase acidic protein
PB1: polymerase basic protein 1
PB2: polymerase basic protein 2
PI3K‐AKT: phophoinositide‐3‐kinase‐protein kinase B
RBD(s): receptor‐binding domain(s)
RdRp: RNA‐dependent RNA polymerase
RER: rough endoplasmic reticulum
RNF43: ring finger 43
RNP: ribonucleoprotein
S: spike
SA: sialic acid
SARS‐CoV‐2: severe acute respiratory syndrome‐coronavirus‐2
Smac: second mitochondria‐derived activator of caspases
ssRNA: single‐stranded RNA
TMPRSS2: transmembrane protease serine 2
TTSP(s): type II transmembrane serine protease(s)
vRNP: viral ribonucleoprotein

## 1 INTRODUCTION

The severe acute respiratory syndrome coronavirus‐2 (SARS‐CoV‐2) emerged as the infectious agent responsible for the coronavirus disease 2019 (COVID‐19) pandemic, and much like the influenza A virus (IAV), it primarily targets the human respiratory system.^1^ Diseases associated with both IAV and SARS‐CoV‐2 infections vary from mild respiratory illness, to acute pneumonia, and even respiratory failure.^2^ Historically, four IAV pandemics have been registered, amongst which the 1918 H1N1 ‘Spanish flu’ was the most devastating, claiming approximately 50 million lives globally.^3^ The recent flu pandemic caused by the H1N1/pdm09 virus, was reported in 2009 in 207 countries and resulted in 42–86 million infections.^4^ This novel IAV has continued to spread as a seasonal epidemic which significantly impacts global health with an annual burden of around 3–5 million cases of severe illness and about 290,000–650,000 mortalities globally as per the world health organization (WHO) reports.^5^ Additionally, a highly pathogenic avian H5N1 IAV has spread throughout Africa, Asia, and Europe by crossing over host species barriers to infect humans and cause mortalities.^6^, ^7^, ^8^, ^9^

Further to the emergencies of SARS caused by a *Betacoronavirus* (SARS‐CoV) in 2002^10^ and Middle East respiratory syndrome (MERS) caused by another type of *Betacoronavirus* (MERS‐CoV) in 2012,^11^ cases of COVID‐19 caused by a new *Betacoronavirus* (SARS‐CoV‐2), were first reported in Wuhan, China in December 2019.^12^ The patients were characterised by acute pneumonia‐associated symptoms, such as chills, dry cough, fever, muscle pain, and shortness of breath.^13^ The SARS‐CoV‐2 rapidly spread globally and as of 11 November 2022, there have been 630,832,131 confirmed cases of COVID‐19, including 6,584,104 deaths, reported to the WHO.^14^ In comparison with the other two coronaviruses, SARS‐CoV‐2 is much more contagious and infectious, which rapidly resulted in a pandemic that constituted a global health emergency.

As of 9 November 2022, a total of 12,885,748,541 anti‐SARS‐CoV‐2 vaccine doses have been administered globally.^14^ These vaccines have been responsible to prevent infection as well as adverse effects caused by several SARS‐CoV‐2 variants, possibly by reducing both asymptomatic and symptomatic infections, thereby preventing the onward transmission and reducing the viral loads.^15^ However, similar viral loads found recently in unvaccinated and vaccinated people infected with various SARS‐CoV‐2 variants has raised questions about the effectiveness of the vaccines to prevent transmission.^16^ Although the vaccines have managed to provide continued protection against infection, the degree of effectiveness varies among the variants, since newer variants, such as delta and omicron tend to erode vaccine‐mediated protection.^17^, ^18^ This challenge necessitated research that focusses on the development of variant‐adapted booster vaccines that can induce higher and broader immune responses against SARS‐CoV‐2.^19^, ^20^, ^21^

Hence, identifying the host cellular targets for treating infections caused by both IAV and SARS‐CoV‐2 is crucial. Host cellular proteases, such as cathepsins and type II transmembrane serine proteases (TTSPs) have been reported to activate SARS‐CoV‐2 to promote their replication. Many members of the B‐cell lymphoma‐2 (Bcl‐2) family proteins are involved in activating these host cellular proteases to promote IAV replication.^22^, ^23^, ^24^, ^25^ In the aftermath of the COVID‐19 pandemic, many recent reviews have focussed on the similarities and differences between these respiratory viruses.^26^, ^27^, ^28^ However, little is known about the role of Bcl‐2 family proteins‐activated proteases in regulating SARS‐CoV‐2 replication. Consequently, this article compares the life cycle and mode of viral replication of IAV and SARS‐CoV‐2 and examines the potential roles of host Bcl‐2 family proteins in SARS‐CoV‐2 replication to provide future research directions.

## 2 STRUCTURE, GENOME, HOST RANGES, AND MODE OF TRANSMISSION

### 2.1 Influenza A virus

IAV is a segmented negative‐sense single‐stranded RNA (ssRNA) virus belonging to the *Orthomyxoviridae* family. It consists of 8 gene segments enclosed in an enveloped virion which is 100 nm in diameter.^29^, ^30^ Haemagglutinin (HA), matrix (M), neuraminidase (NA), nucleoprotein (NP), non‐structural protein (NS1), polymerase acidic protein (PA), polymerase basic protein 1 (PB1), and polymerase basic protein 2 (PB2) are the eight IAV gene segments which encode protein(s) with specific functions.^31^, ^32^ The segmented genome of IAV has important implications in virus evolution and immune escape.^33^

The IAVs are divided into subtypes based on the sequences of HA and NA that are located on the surface of the virion envelope.^32^ At least 18 HA types (H1 to H18) and 11 NA types (N1 to N11) have been identified so far.^34^ IAVs infect various animal species such as birds, dogs, ferrets, horses, and pigs. Although, the main viral reservoirs, aquatic birds like waterfowls remain asymptomatic, IAVs pose a threat to humans as a zoonotic agent, generally causing mild infections.^35^, ^36^, ^37^ However, some specific subtypes, like H1N1 and H5N1, cause severe illnesses/mortality in infected patients.^38^ Further, crossover to other animals may lead to the emergence of pathogenic subtypes, such as the 1968 Hong Kong flu pandemic (H3N2) and the 2009 swine flu pandemic (H1N1) that caused severe disease.^39^ IAVs that infect humans mainly belong to H1N1 and H3N2 sub‐types.^40^ IAVs causes both upper and lower respiratory tract infections and may attack other organs such as the central nervous system (CNS) and heart.^41^ The mode of transmission of IAVs is mainly by respiratory droplets. Three routes of transmission (not mutually exclusive) have been postulated and are widely accepted: contact transmission (direct/indirect), large droplets, and respiratory droplets (or aerosols) albeit the relative contribution of each to the rapid spread and continued circulation of IAV is highly debatable. For IAV to cause infection in a host, a variety of factors related to the environment, the host, and the virus may play a role.^42^

IAVs exhibit antigenic drift/shift properties, genetic phenomenon allowing them to avoid the host immune response via mutations.^27^ Antigenic drift is the variation that occurs in antigen structures owing to point mutations in the HA and NA genes over time due to the lack of proofreading by viral RNA‐dependent RNA polymerase (RdRp) usually leading to seasonal differences and epidemics. Antigenic shift, on the other hand, is the result of a sudden genetic reassortment between genome segments, which leads to the formation of a totally new virus and often leads to pandemics.^43^ These antigenic drift/shift properties can potentially reduce the effectiveness of vaccines and become a considerable challenge in antiviral therapy.^44^ Antiviral drugs like amantadine/rimantadine,^45^ oseltamivir, and zanamivir^40^ are used for prophylaxis and treatment of IAV infection; however, most human H1N1 and H3N2 viruses, including the 2009 H1N1 pandemic IAV, are resistant to these drugs and the frequency of resistance is increasing with time.^46^, ^47^, ^48^, ^49^ Consequently, currently vaccination is the most effective way to prevent IAV outbreaks. Unfortunately, high incidences of mutation in both the HA and NA makes complete protection difficult. Presently, a universal flu vaccine that will be effective against any new IAV strain is the focus of flu researchers and hopefully, if achieved, will put an end to the yearly seasonal flu outbreaks.^50^

### 2.2 SARS‐CoV‐2

Coronaviruses (CoVs) are a group of viruses belonging to the *Coronaviridae* family and the *Orthocoronavirinae* subfamily.^51^, ^52^ The CoV genome has the form of positive‐sense ssRNA encased in an enveloped helical nucleocapsid with the virion of approximately 125 nm diameter.^51^, ^52^ SARS‐CoV and SARS‐CoV‐2 are the two highly pathogenic members of the CoV group. SARS‐CoV is characterised by the ability to cause SARS in infected humans and led to a global outbreak of pneumonia resulting in approximately 800 deaths in 2003.^53^ Studies on human and mammalian cell lines have demonstrated a SARS‐CoV‐induced cytopathic effect which exhibits caspase activation and typical apoptotic morphology,^54^ suggesting that like IAVs, apoptosis may be required for the SARS‐CoV replication.^55^, ^56^

SARS‐CoV‐2 is the etiological agent of COVID‐19^57^ that emerged in December 2019 as the infectious agent causing a new respiratory disease.^58^ On 11 March 2020, the WHO labelled its outbreak a pandemic, and the status of the disease has not changed much since then.^59^, ^60^, ^61^ The RNA genome size of SARS‐CoV‐2 is 30,000 bases in length. Among other betacoronaviruses, SARS‐CoV‐2 is characterised by a unique combination of polybasic cleavage sites, a distinctive feature known to increase pathogenicity and transmissibility in other viruses.^62^

Genomic analysis of SARS‐CoV‐2 revealed that the genome comprises 14 open reading frames (ORFs), two‐thirds of which encode 16 nonstructural proteins (nsp 1–16) that make up the replicase complex.^63^, ^64^ The remaining one‐third encodes 9 accessory proteins (ORFs) and 4 structural proteins; envelope (E), membrane (Memb), nucleocapsid (N), and spike (S). The SARS‐CoV‐2 genome is encapsidated by N, whereas M and E ensure its incorporation in the viral particle during the assembly process. S trimers protrude from the host‐derived viral envelope and provide specificity for cellular entry receptors and have been shown to mediate viral entry into the host cells.^65^ However, SARS‐CoV‐2‐S is highly variable from SARS‐CoV, sharing <75% nucleotide identity.^13^, ^66^, ^67^

## 3 LIFE CYCLE, MECHANISM OF REPLICATION AND PATHOGENESIS

### 3.1 Influenza A virus

**FIGURE 1:**
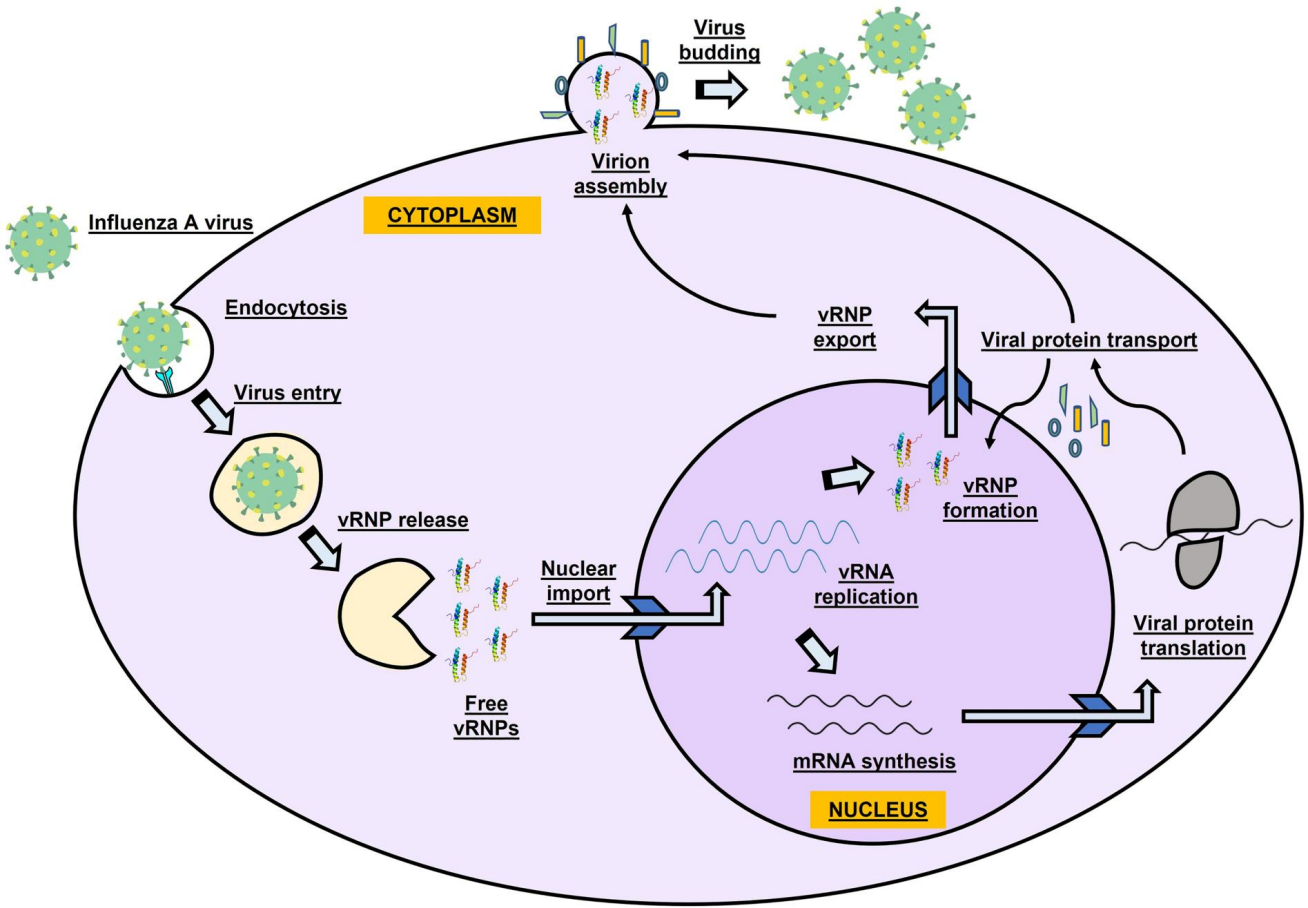
Schematic representation of the life/replication cycle of influenza A virus (IAV). The IAV haemagglutinin binds to sialylated glycoprotein receptors on the cell surface leading to viral endocytosis. Following the fusion of viral and endosome membranes, the viral genome is then released into the cytoplasm. The viral ribonucleoproteins (vRNPs) are imported into the nucleus leading to viral transcription and replication. Further, translation of viral proteins occurs in the cytoplasm and some viral proteins are even transported back to the nucleus. Finally, the progeny virions are assembled and exocytosed from the cell surface by budding.

The life cycle of IAV is generally divided into four steps: virus entry into the host cell, transcription and replication of the viral genome, assembly, and virus budding (Figure 1). For human IAVs, type 2 alveolar epithelial cells (AEC2s), as well as the immune cells such as alveolar macrophages and dendritic cells, are the primary targets for an established infection.^68^, ^69^ IAV infection starts from recognition of sialic acid (SA) by HA protein, though in vitro studies suggest that these N‐linked glycans are not essential for virus entry.^70^ HA facilitates attachment and viral genome is uncoated and transported to the nucleus where genome segments are transcribed by viral RdRp into positive‐sense mRNA and translated into the various non‐structural and structural proteins needed for IAV replication (Figure 1). TTSPs‐like transmembrane protease serine 2 (TMPRSS2) and TMPRSS4 may be involved in cleaving avian and human IAV HA proteins at an arginine residue which is essential for proteolytic activation of IAV HA subtypes.^71^ Cleavage of the envelope glycoprotein HA by host proteases is a prerequisite for membrane fusion and essential for virus infectivity.^72^ This suggests that TMPRSS2 may be a potential target for treatment of both influenza and coronavirus infections. Proteins in the viral ribonucleoprotein (vRNP) complex contain different nuclear localisation signals, thus helping the vRNP complex to enter the host cell nucleus via active transport.^73^ The acidic environment of the endosome also activates M2 ion channel, hence acidifying the viral core, resulting in entrance of vRNP complex into the host cell.^74^ Replication of viral genome does not require a primer but a full‐length complementary RNA (cRNA), which is essential for the newly formed vRNP complex. The viral RNA polymerases first bind to the 3ʹ and 5ʹ‐end of the segmented viral RNA and cRNA, respectively, then starts replication with the help of the 5ʹ cap of host pre‐mRNAs via a PB1–PB2‐mediated ‘cap snatching’ mechanism.^75^ The conserved segment‐specific nucleotides at the 3ʹ and 5ʹ ends of the viral genome can modulate genome expression and replication during infection.^76^

Mature viral mRNAs are transported to the cytoplasm by a ‘daisy‐chain’ complex and translated subsequently.^73^, ^77^ New synthesis of HA occurs on the rough endoplasmic reticulum. After synthesis and maturation of NA and M2 proteins, the trans‐golgi network, together with the coat protein I complex and the Rab GTPase proteins, transports the newly synthesised HA, M2, and NA proteins to the apical plasma membrane where they assemble with the viral genomic segments. The virions are finally closed and M1 and M2 proteins mediate virion budding from the apical side of the cells.^78^, ^79^, ^80^ New IAV virions bud out of the cell by the help of NA, which cleaves SA from mucins and cell surfaces^81^ (Figure 1). PA, PB1, and PB2 polymerase proteins form an enzyme complex that plays a role in transcription and replication. The NP is used as a model to generate additional copies.^82^ Conceptually, any of the above discussed steps in the IAV replication cycle could potentially serve as an antiviral drug target,^83^, ^84^, ^85^ but the frequent evolution of IAVs has contributed to the development of drug resistance. Thus, modulating the host factors responsible for viral replication could be a better strategy against IAVs.

### 3.2 SARS‐CoV‐2

Epithelial cells in the nasal cavity are the primary site of attachment and replication of the inhaled SARS‐CoV‐2. The infectious agent then spreads and migrates across the respiratory tract, following the conducting airways.^86^ The coronavirus invades two types of cells in the lungs, the mucus producing goblet cells and the ciliated cells. The cilia cells are the preferred viral host.^87^ Coronavirus spike protein interacts with cellular receptors, angiotensin‐converting enzyme 2 (ACE2), found in human cells in the heart, intestines, kidneys, and lungs.^88^ The cell surface serine protease, TMPRSS2, promotes viral uptake and fusion at the cellular or endosomal membrane. SARS‐CoV‐2‐S contains two receptor‐binding domains (RBDs), S1 and S2, that mediates direct contact with ACE2, and an S1/S2 polybasic cleavage site that is proteolytically cleaved by cellular cathepsin L and TMPRSS2.^66^, ^67^, ^89^ RBDs are used to bind with the ACE2 receptors, allowing the virion to penetrate eventually into the human cells by a process known as endocytosis.^90^ TMPRSS2 facilitates viral entry at the plasma membrane surface, whereas cathepsin L activates SARS‐CoV‐2‐S in endosomes and can compensate for entry into cells that lack TMPRSS2.^89^

**FIGURE 2:**
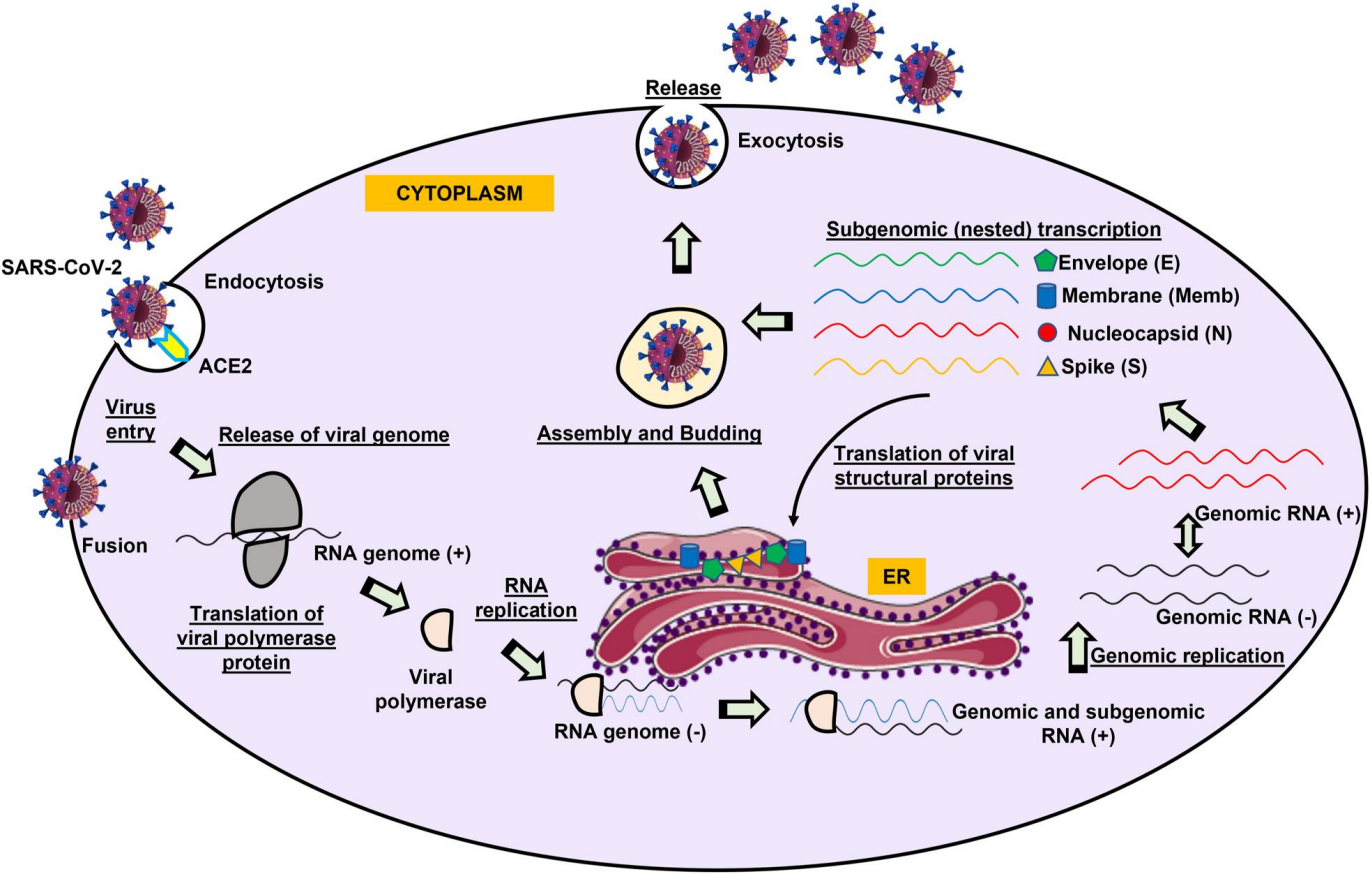
Schematic representation of the life/replication cycle of severe acute respiratory syndrome coronavirus‐2 (SARS‐CoV‐2). SARS‐CoV‐2 binds to its cognate receptor, angiotensin‐converting enzyme 2 (ACE2), by specific interactions involving the viral envelope spike protein, which is followed by the virus‐cell membrane fusion at the cell surface and endosomal compartments, and release of the RNA genome into the cytosol. The assembly of viral replication‐transcription complexes leads to viral RNA synthesis which translates to viral structural proteins that are further inserted into the endoplasmic reticulum (ER). Genomic RNA is packaged into ribonucleoprotein complexes and virions are then assembled and transported out of the infected cells by exocytosis.

Once inside the cytoplasm, SARS‐CoV‐2 dissolves its own protein shells and releases the viral RNA payload inside the cell (Figure 2). The viral RNA hijacks the cell replication machinery and starts to replicate itself. It also manufactures the necessary proteins to assemble new viruses, that the golgi bodies transport out of the infected cell by a process called exocytosis. In a nutshell, following entry, the release (into the host cytosol) and uncoating of the incoming genomic RNA, subject it to the immediate translation of two large ORFs, ORF1a and ORF1b. The resulting viral replicase polyproteins, pp1a and pp1ab, are co‐translationally and post‐translationally processed into the individual nsps (via host and viral proteases) that form the viral replication and transcription complex.^65^ Further, rearrangement of the endoplasmic reticulum (ER) into double‐membrane vesicles occurs which facilitates viral replication of genomic and sub‐genomic mRNAs; the latter are translated into accessory and viral structural proteins that facilitates virus particle formation.^91^ Translated structural proteins translocate into ER membranes and transit through the ER‐to‐golgi intermediate compartment, where interaction with N‐encapsidated newly produced genomic RNA results in budding into the lumen of secretory vesicular compartments. Finally, virions are secreted from the infected cell by exocytosis (Figure 2).

## 4 APOPTOSIS IN VIRAL PATHOGENESIS

### 4.1 Influenza A virus

IAVs can facilitate the progress of its pathogenesis and transmission by inducing apoptosis in the airway epithelial cells,^24^ natural killer cells,^92^ and neutrophils.^93^ Several viral proteins are involved in the regulation of host apoptosis. As viral replication must be completed before dismantling of the cell through apoptosis, the expression of the anti‐apoptotic viral proteins may facilitate viral propagation prior to cell death. Activation of the apoptotic cascade has been shown to play a role in the processing of viral proteins and the maturation of viral particles.^94^ The pro‐apoptotic proteins promote IAV‐induced apoptosis and virus replication^95^, ^96^, ^97^ by facilitating the proper shuttling of viral genomic segments from the nucleus to the budding sites at the plasma membrane. In line with this, the multifunctional IAV protein NS1 has been implicated in suppressing the host interferon response,^98^ and both promoting^99^ and inhibiting^100^ apoptosis. Although the precise mechanism of how NS1 can limit apoptosis is not fully understood, it is quite possible that NS1 directly interacts with and inhibits pro‐apoptotic host factors through a N‐terminal PDZ‐binding motif.^100^ It is interesting to note that the PDZ‐binding motif of NS1 is also required for efficient viral propagation, as mutation of this domain can significantly reduce viral titres.^100^ Hence, NS1 may prevent the early induction of apoptosis, consequentially aiding IAV pathogenesis.

Furthermore, IAV NP has been reported to induce host apoptosis to favour viral replication through interaction with ring finger 43 (RNF43) and apoptotic inhibitor 5 (API5)^101^ or clusterin.^102^ Even though the mechanism of NP‐induced apoptosis is not fully elucidated yet, past reports have shown that NP can directly interact with the anti‐apoptotic host factor API5, thereby preventing downregulation of APAF1 and promoting apoptosis.^103^ NP exerts its pro‐apoptotic function through the inhibition of the E3 ubiquitin ligase RNF43.^101^ As RNF43 can mark p53 for degradation through ubiquitination, the interaction of NP with RNF43 can result in p53 stabilisation and consequently promote apoptosis through Bak/Bax activation.^101^ Further, the IAV M1 has been reported to promote apoptosis by binding to heat shock protein 70, thus, activating the caspase cascade followed by apoptosis.^104^ A recent transcriptomic study highlighted that apoptosis marker genes were expressed early in infection, at 8 h post infection (hpi), which is a host strategy to limit viral infection. The IAV PB1‐F2 gene was suggested to be responsible for this induction.^105^ PB1‐F2 induced apoptosis and promoted viral replication through dysregulating mitochondrial potential.^106^ Conclusively, these studies highlight the pro‐viral role of apoptosis during the pathogenesis of IAV.^107^ The role of these viral proteins in apoptosis suggests that they might be suitable targets for anti‐IAV therapies.^63^

### 4.2 SARS‐CoV‐2

Recent novel studies on SARS‐CoV‐2 provide valuable insights into the possible role of cell death in the infection‐induced tissue injury.^56^ Apoptosis induction has been labelled as a hallmark of SARS‐CoV‐2 infection.^108^ SARS‐CoV‐2 induces the apoptosis of the infected alveolar cells by using human lung stem cell‐based alveolospheres.^109^ Extensive apoptosis has been observed in lung epithelial cells of humanised ACE2 transgenic mice and Syrian hamsters.^34^ Interestingly, the expression of ORF3a of SARS‐CoV‐2 is less cytotoxic than that of SARS‐CoV and it stimulates less apoptosis and caspase activation in human cell lines.^110^ This observation explains why SARS‐CoV‐2 exhibits lower case‐fatality rate than SARS‐CoV (∼10%). Conversely, direct SARS‐CoV‐2 infection of organotypic airway epithelial cells results in higher cytopathic effect with apoptotic characteristic as compared with infection with human coronavirus (HCoV)‐NL63, a less pathogenic member among the HCoVs.^111^

Interesting novel findings support the hypothesis that NLRP3 inflammasome is activated in response to SARS‐CoV‐2‐S/ACE2 receptor binding in haematopoietic and endothelial cells.^112^ The ion channel property of SARS‐CoV‐2‐E could potentially serve as an important factor in this context. Past reports have suggested the possible role of Bcl‐xL in the pathogenesis of SARS.^113^ A feature of the disease is the substantial loss of host lymphocytes that leads to lymphopenia; hence, Jurkat T cells were used to examine the pro‐apoptotic ability of SARS‐CoV. The cells were transfected with cDNA encoding viral envelope protein. SARS‐CoV‐E protein increased the level of apoptosis, indicating the possible reason for the SARS‐associated lymphopenia. Other studies revealed that Bcl‐xL overexpression prevented the SARS‐CoV‐E‐induced death of Jurkat T cells, and that SARS‐CoV‐E interacts with Bcl‐xL through the BH3‐like motif of SARS‐CoV‐E and the BH3 domain of Bcl‐xL.^113^

Apoptosis induced by infection of MERS‐CoV, SARS‐CoV, or other HCoVs was described in various in vitro systems and animal models and can be induced by multiple mechanisms.^56^, ^114^ SARS‐CoV induces caspase‐dependent apoptosis,^55^ which is dependent on, but not essential for viral replication, as treatment with pan‐caspase inhibitor z‐VAD‐FMK or overexpression of Bcl‐2 did not significantly affect SARS‐CoV replication.^115^ Recently, SARS‐CoV‐2 infection was reported to activate caspase‐8 resulting in apoptosis induction and inflammatory response in the lung epithelial cells.^116^ In contrast, while MERS‐CoV infection of human primary T lymphocytes was abortive, apoptosis was induced via activation of both intrinsic and extrinsic pathways.^117^ Apoptosis in neuronal cells infected with HCoV‐OC43 involved mitochondrial translocation of Bax but was independent of caspase activation.^118^ HCoV infection also modulated apoptosis by activating ER stress response and mitogen‐activated protein kinase pathway. Studies have shown that apoptosis can be induced by SARS‐CoV proteins.^119^ Other pro‐apoptotic mechanisms by SARS‐CoV include interfering with pro‐survival signalling by M protein and the ion channel activity of SARS‐CoV‐E and 3a proteins.^119^

**FIGURE 3:**
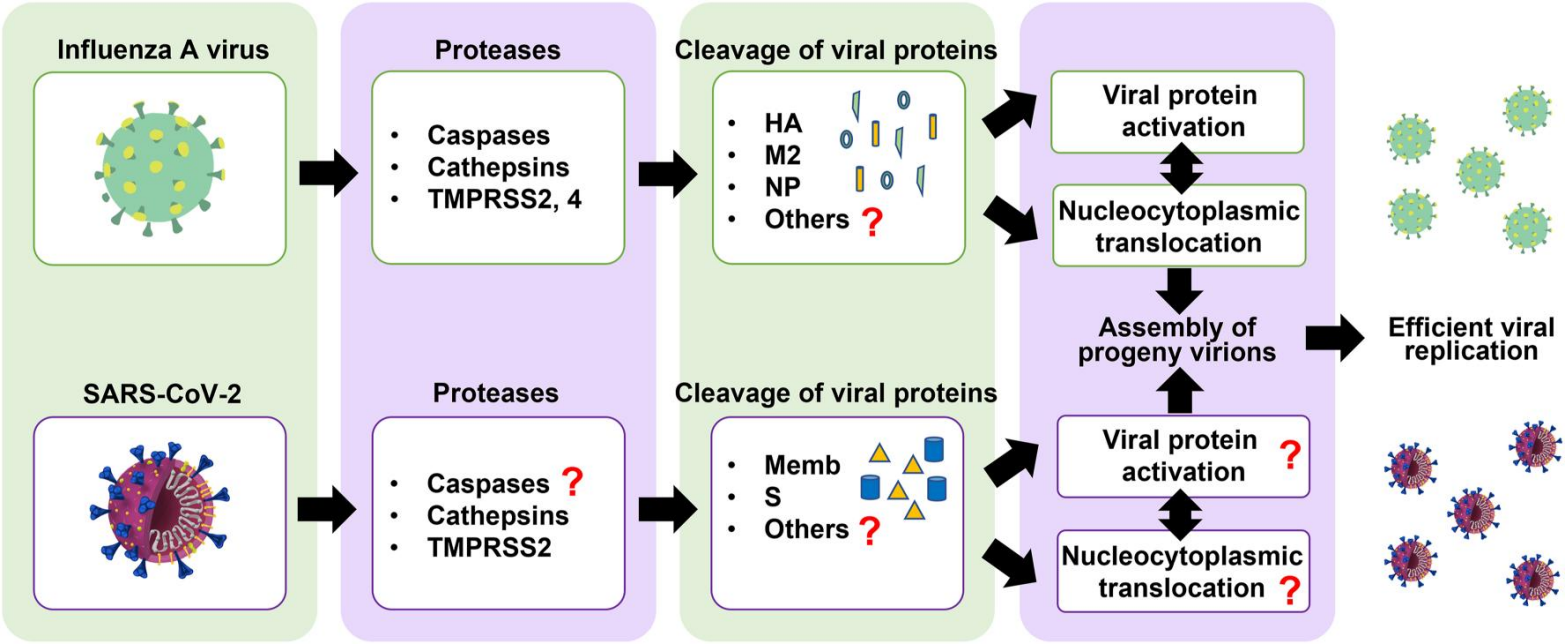
Schematic representation of the role of proteases in viral replication cycle. Proteases such as caspases, cathepsins, transmembrane protease serine 2 (TMPRSS2), and TMPRSS4 have been reported to cleave and activate influenza A virus (IAV) proteins (matrix 2 [M2] and nucleoprotein [NP]), and aid in efficient nucleo‐cytoplasmic shuttling resulting in efficient viral replication. Similarly, proteases, such as cathepsins and TMPRSS2 cleave severe acute respiratory syndrome coronavirus‐2 (SARS‐CoV‐2) proteins (membrane [Memb] and spike [S]) which might lead to activation of viral proteins and/or efficient nucleo‐cytoplasmic shuttling of components of the viral proteins leading to proper assembly and efficient viral replication.

Emerging studies propose that SARS‐CoV‐2 infection‐associated epithelial cell apoptosis plays a vital role in lungs disorder.^120^ SARS‐CoV‐2 membrane protein induces lung epithelial cells mitochondrial apoptosis by stabilising Bcl‐2 ovarian killer (Bok) via inhibiting its ubiquitination and promoted Bok mitochondria translocation. SARS‐CoV‐2‐M interacted with Bok through its endodomain. Bok^−/−^ by CRISPR/Cas9 increased cellular resistance to SARS‐CoV‐2‐M‐induced apoptosis. Interestingly, SARS‐CoV‐2‐M induced Bok to trigger apoptosis in the absence of Bak and Bax. Furthermore, the BH2 domain of Bok was required for interaction with SARS‐CoV‐2‐M protein and pro‐apoptosis. In an in vivo model, SARS‐CoV‐2‐M recombinant lentivirus infection was shown to induce caspase‐associated apoptosis and increased alveolar‐capillary permeability in the mouse lungs and BOK knockdown improved the lung oedema due to lentivirus‐M protein infection^116^ (Figure 3). These findings suggest a pro‐apoptotic role of SARS‐CoV‐2‐M in viral pathogenesis and point to the fact that a failure of apoptosis activation would hamper SARS‐CoV‐2 clearance from infected cells. Hence, targeting virus‐induced apoptosis might prove to be a potential strategy in the treatment of SARS‐CoV‐2 virus infection.

## 5 THE BCL‐2 FAMILY PROTEINS AND THEIR ROLE IN APOPTOSIS

The Bcl‐2 family proteins are critical regulators of cell survival and apoptotic cell death. The pro‐survival or the anti‐apoptotic members include Bcl‐2, Bcl‐W, Bcl‐xL, and Mcl‐1 and contain up to four shared Bcl‐2 homology (BH) domains. The pro‐apoptotic proteins are divided into multi‐BH domain members, such as Bak and Bax, containing BH1‐3 domains, and the ‘BH3‐only’ members like Bad, Bid, Bcl‐2 interacting killer (Bik), and Bim.^121^ Pro‐apoptotic BH3‐only proteins are upstream mediators of cell death, and their pro‐apoptotic activity is tightly regulated by diverse transcriptional and post‐transcriptional mechanisms.^122^

The apoptotic process can be activated by two major pathways, the intrinsic or the mitochondrial pathway orchestrated by the Bcl‐2 family proteins and the extrinsic or the death receptor pathway regulated by the extracellular ligands and receptors.^123^, ^124^ Following apoptotic stimulation by stress signals, active forms of the pro‐apoptotic pore‐forming multi‐BH domain members of Bcl‐2 family proteins (Bak and Bax) generates pores within the outer mitochondrial membrane (OMM), hence destabilising it and promoting mitochondrial outer membrane permeabilisation.^125^ The mitochondrial intermembrane space proteins like cytochrome c, second mitochondria‐derived activator of caspases (Smac)/direct inhibitor of apoptosis‐binding protein with low pI (isoelectric point) (DIABLO) and other pro‐apoptotic factors are released into the cytosol which triggers the activation of the caspase cascade and apoptotic cell death.^126^ Cytochrome c forms a complex called apoptosome by binding to the apoptotic protease activating factor‐1 (Apaf‐1) in the cytosol which initiates caspase activation cascade. Activated caspase‐9 cleaves and activates caspase‐3 to cause apoptosis.^127^, ^128^ Certain stresses like DNA damage and deprivation of growth factors activate the BH3‐only proteins which function to negate the inhibitory effect of the pro‐survival Bcl‐2 family proteins. Further, the anti‐apoptotic Bcl‐2 family proteins, such as Bcl‐2 and Bcl‐xL interact with Bak or Bax causing their inactivation and thereby preventing mitochondrial permeabilisation.^129^, ^130^ Various viruses encode proteins that interact with apoptosis‐regulating proteins,^131^ although the effects of such interactions on virus multiplication are largely unknown.

During infection, IAV proteins interact with host cellular proteins and use the cellular machinery for virus replication. Host cell death induction is essential for efficient replication of IAVs and is linked to IAV pathogenesis.^132^, ^133^, ^134^ While increased expression of the pro‐survival Bcl‐2 protein impairs IAV replication,^135^, ^136^ the pro‐apoptotic proteins such as Bad,^23^ Bax,^95^ and Bik^24^ promotes virus replication by activating host cellular proteases that cleave and activate viral proteins^24^ and/or promotes nucleo‐cytoplasmic shuttling of viral proteins.^24^

## 6 BCL‐2 FAMILY PROTEINS AND IAV REPLICATION

Past studies have indicated that the anti‐apoptotic Bcl‐2 family proteins are involved in promoting viral replication at an early stage of infection.^137^ For example, in IAV‐infected retinal pigment epithelium cells, the interaction of Bcl‐2, Bcl‐w, and Bcl‐xL with Bad/Bax counteracted the OMM permeabilisation, thwarted the consequent release of pro‐apoptotic factors from the mitochondria to the cytosol, and prevented the downstream signalling cascade. The anti‐apoptotic proteins seem to delay the intrinsic apoptosis induction and maintain cell viability at the onset of infection to enable the synthesis of progeny vRNPs in the nucleus. After IAV infection, the vRNPs are transported to the host cell nucleus, where they undergo transcription and replication. In the late phase of replication, newly formed RNPs are transferred from the nucleus to the cytoplasm and packaged into progeny virions.^73^, ^138^ This intrinsic step in the life cycle of the virus is known to be regulated to a certain extent by the viral and host cell factors,^96^, ^139^ including the expression of Bcl‐2, which varies widely from one cell type to another.^136^ Although expression of Bcl‐2 does not protect host cells from virus‐induced apoptosis, several lines of evidence indicate that host cell expression of Bcl‐2 is associated with both the reduction in virus replication^135^, ^136^ and impairment of nucleo‐cytoplasmic translocation of vRNPs.^136^ The latter effect might be related to interference of Bcl‐2 with one or more cellular phosphorylation pathways, since phosphorylation events are known to play crucial roles in the regulation of vRNP traffic.^139^, ^140^

IAV‐induces apoptotic cell death mediated by the Bcl‐2 family proteins in a variety of host cells including epithelial cells, macrophages, and monocytes, under both in vitro and in vivo conditions.^23^, ^141^, ^142^ Interestingly, some viruses inhibit host cell apoptosis with several mechanisms, including the expression of Bcl‐2 homologues. By preventing the premature death of its host, the virus allows itself to replicate and to establish persistent infections. On the contrary, other viruses induce apoptosis of infected cells by several mechanisms, including Bcl‐2 destabilisation leading to viral dissemination.^106^ It appears that IAV has evolved strategies to modulate apoptosis to promote efficient virus replication. During IAV infection, viruses have been shown to modulate host apoptotic responses in a time‐dependent manner.^106^ To earn enough time for replication and virion formation, IAVs have been reported to inhibit apoptosis via upregulating the anti‐apoptotic phophoinositide‐3‐kinase‐protein kinase B (PI3K‐AKT) pathway at the onset of infection. In the later phase of infection, the virus suppressed this pathway to promote apoptotic and/or necrotic pathways such as p53 pathway, thus, accelerating death of infected cells and allowing successful release of virions.^142^, ^143^, ^144^ A past study showed that apoptosis was initiated almost 24 hpi and was marked by activation of caspase‐3, ‐7, and ‐9 and subsequent cleavage of PARP‐1 and truncation of Bid. These events were accompanied by increased p53 expression during culmination of infection (18–24 hpi),^145^ consistent with other reports on apoptotic cell death during IAV infection.^24^

Several studies have highlighted the importance of apoptosis induced by IAV using different strains and subtypes of IAV, such as A/Cal/07/2009 and A/PR/8/1934 (H1N1)^23^; A/Wenshan/01/2009 (H1N1),^146^ and A/NY/55/2004 (H3N2)^23^ and have investigated the role of Bcl‐2 family proteins in IAV infection.^24^, ^95^, ^147^ IAV‐induced apoptosis is shown to be dependent on Bak/Bax activity^95^ through downregulation of anti‐apoptotic factors such as Bcl‐xL and Mcl‐1.^147^ Deletion of other pro‐apoptotic Bcl‐2 family proteins, Bak and Bax, also results in increased PR/8 replication. Although an antagonistic effect of Bcl‐2 family proteins in IAV replication is reported, several lines of evidence indicate otherwise.^95^, ^135^ Unlike other pro‐apoptotic members, Bak may have antiviral effects as its expression is significantly downregulated during IAV infection and its expression suppresses IAV replication. However, Bax activation at 24 hpi, enhanced virus propagation and Bax‐deficiency prevented IAV‐induced apoptosis and caused diminished viral replication.^95^ This suggests that although both Bak and Bax are major apoptotic proteins, they have opposing roles in IAV replication.

## 7 BCL‐2 FAMILY PROTEINS‐ACTIVATED HOST CELLULAR PROTEASES AND THEIR ROLE IN IAV AND SARS‐COV‐2 REPLICATION

Several viruses, including IAVs, express proteins that undergo host cell caspase cleavage.^148^ IAV‐induced caspase activation causes a widening of nuclear pores to facilitate passive transport of vRNP to ensure efficient production of infectious virus progeny.^149^, ^150^ IAVs are structured into RNP segments consisting of viral RNA and viral proteins, the major one being the NP, a target of caspase cleavage.^24^, ^151^ NP acts as a shuttle for viral genomic segments from the nucleus to the budding sites at the plasma membrane, and localisation during the virus replication cycle affects virus titres. Following apoptotic stimuli, IAV NP or RNP has been shown to translocate partially to the cytoplasm in a caspase‐3‐dependent manner,^96^ and nuclear retention of NP caused by a lack of caspase activity has been linked to decreased viral replication.^136^ The pro‐survival protein Bcl‐2, on the other hand, inhibits IAV‐induced apoptosis to mitigate viral replication rates.^135^, ^152^ Activation of caspase‐3 during the cell death process is critical to the IAV life cycle^96^ and increased expression of Bcl‐2 blocks caspase activation and leads to improper nuclear retention of IAV RNP complexes that causes improper assembly of progeny virions and marked reduction in titres of infectious virus.^24^, ^96^, ^151^, ^152^ Possibly, IAVs use a strategy where caspases regulate vRNP export by increasing the diffusion limit of nuclear pores to allow passive diffusion of vRNP.^149^

Our group found a link between Bik‐mediated caspase activation and cleavage of viral proteins. IAV replication was diminished in mouse airway epithelial cells (MAECs) isolated from bik^−/−^ as compared with bik^+/+^ mice. bik^−/−^ MAECs showed more stable transepithelial resistance after infection, were less sensitive to infection‐induced cell death, and released fewer copies of viral RNA compared with bik^+/+^ MAECs. We found similar results when Bik expression was suppressed in human airway epithelial cells (HAECs). More interestingly, bik^−/−^ mice were protected from IAV‐induced mortality and were 10‐fold less likely to die from infection as compared with bik^+/+^ mice. Mechanistically, IAV infection activated caspase‐3 in bik^+/+^ but not in bik^−/−^ MAECs. Furthermore, cleavage of viral M2 and NP proteins were inhibited in HAECs when Bik expression was suppressed and in cells in which caspase activation was blocked by a pan‐caspase inhibitor, Q‐VD‐OPh. This study suggested a link between Bik‐mediated caspase activation and cleavage of viral proteins supporting the hypothesis that Bik‐mediated caspase cleavage of viral protein(s) promotes IAV replication. Bik‐deficiency also impaired cytoplasmic export of vRNP, suggesting the potential for impaired shuttling of vRNP to the cytoplasm by Bik‐deficiency.^24^ It is not clear, however, whether the defects in the cleavage of viral proteins are caused by Bik‐deficiency or increased expression of Bcl‐2 contributes partially to the nuclear retention of viral NPs. Similarly, the viral ionic channel M2 protein is also cleaved by caspases in both human and avian influenza viruses^153^, ^154^ (Figure 3). Earlier studies also investigated the role of Bad, Bak, and Bax in IAV infection.^95^, ^135^ BAD promotes IAV replication through phosphorylation of BAD at S112 and S136 and apoptotic death of epithelial cells.^23^ Bax and Bak are also involved in IAV replication and virus‐induced cell death. Bak is downregulated during IAV infection, and Bak^−/−^ yields a rise in the rate of apoptotic death with a corresponding increase in levels of virus replication, suggesting that Bak suppresses both apoptosis and viral replication.^95^ Contrary to Bak, downregulation of Bax blocks the induction of apoptosis, restricts the infection‐mediated activation of executioner caspases, and inhibits virus propagation. Furthermore, Bax^−/−^ causes a retention of IAV NP within the nucleus, suggesting that Bax‐mediated caspase activation may be involved in the nucleo‐cytoplasmic shuttling of vRNP.^95^ Currently protease inhibitors have been used as effective tools to target hepatitis C virus (HCV) and HIV‐1 infections. HIV‐1 protease inhibitors are effective antiretroviral drugs that prevent cleavage of HIV‐1 viral proteins, resulting in superior antiviral activity.^155^ The HCV specific protease inhibitors block the enzymatic activity of the HCV non‐structural protein 3 (NS3) protease region that is necessary for protein processing required for viral replication. At least four HCV protease inhibitors have been approved for use in the United States (boceprevir, glecaprevir, grazoprevir, paritaprevir, simeprevir, telaprevir) and the combination of an HCV protease inhibitor with other antiviral agents (such as peginterferon, ribavirin, and the direct acting agents that block the NS5A and NS5B regions of the virus) leads to marked decrease in HCV replication and eradication of the infection in a high proportion of patients with chronic hepatitis C.^156^, ^157^ Antiviral drugs and vaccine approaches for IAV and SARS‐CoV‐2 that are presently being used clinically or undergoing trials have been reported (reviewed in Ref.^26^). However, none of these antiviral approaches target Bcl‐2 family proteins. Hence, IAV and/or SARS‐CoV‐2 protein‐activating host cell caspases may provide novel potential drug targets.^55^ Inhibition of host factors (such as NP or M2‐activating caspases) either by modulating the pro‐apoptotic proteins (such as the Bik level) or by blocking the activity of specific caspases could be a novel approach to mitigate IAV replication (Figure 3).

## 8 PERSPECTIVES AND FUTURE DIRECTIONS

Both IAV and SARS‐CoV‐2 are enveloped RNA viruses and appear to have similar mode of infection, molecular mechanisms of replication, and transmission (reviewed in Ref.^27^). The molecular mechanisms of IAV replication and pathogenesis have relatively been well explored. Owing to the many similarities between these infectious agents, important lessons can be learnt from the knowledge available so far on IAV to lay a fundamental basis of understanding for SARS‐CoV‐2. Antiviral drugs such as amantadine/rimantadine,^45^ oseltamivir, and zanamivir,^40^ are used for prophylaxis and treatment of IAV infection; however, most human H1N1 and H3N2 viruses, including the 2009 pandemic H1N1 IAV, are resistant to these drugs and the frequency of resistance is increasing steadily.^46^, ^47^, ^48^, ^49^ Although efforts are underway to find effective treatment for SARS‐CoV‐2, the recent appearances of new variants, such as delta^158^ and omicron^159^ suggests that, similar to IAVs, SARS‐CoV‐2 could continue to mutate and become resistant to the available drugs. Therefore, a shift from current targets and strategies towards development of treatments or vaccines that work for all strains and reduce the costs and burden of disease will be of great importance. Cellular factors that interact with viral proteins to enhance or inhibit virus replication may impact the replication of all strains. These include interaction blockers of viral proteins with host cell proteins. Deciphering the host factors that promote or inhibit virus replication can provide important insights into viral pathogenicity, paving a way for the development of new antiviral drugs that work for multiple emerging strains of SARS‐CoV‐2. Thus, the treatment and containment of SARS‐CoV‐2 pandemic over the next decade will largely depend on detailed understanding of the life cycle, virus‐host cell interaction, and mechanism of virus replication. Vigorous research and investigation that focuses on possible viral polymorphisms, mutations, and angle of evolution would be invaluable.

Inhibitors of host cell proteases have been used effectively to reduce viral replication and treat viral infections, such as in the case of HCV^157^ and HIV‐1.^155^ The recent breakthroughs that led to the development of protease inhibitors, such as grazoprevir and elbasvir that are effective in treating hepatitis C^156^, ^157^ suggests that host cellular proteins can be good targets to treat infections caused by IAV and/or SARS‐CoV‐2. Host cellular proteases, such as caspases, cathepsins, and transmembrane proteases, such as TMPRSS2 have been implicated in the replication of both IAV and SARS‐CoV‐2 through cleaving and activating viral proteins to promote their replication.^24^, ^148^, ^160^ For example, TMPRSS2 cleaves HA protein of IAVs and spike protein of SARS‐CoV‐2 to activate and promote their replication, suggesting that TMPRSS2 may be a potential target for the treatment of both influenza and coronavirus infections. In the case of IAVs, the pro‐apoptotic members of the Bcl‐2 family proteins mediate activation of caspases and cathepsins^24^, ^148^ that causes nuclear pore opening,^161^ to allow efficient nucleocytoplasmic shuttling and proper assembly of components of viral proteins.^24^ However, little is known whether members of the Bcl‐2 family proteins are involved in activating TMPRSS2 and whether proteases activated by the pro‐apoptotic members of the Bcl‐2 family proteins, such as caspases and cathepsins, have role in SARS‐CoV‐2 protein's cleavage and activation. Several studies have identified the role of the Bcl‐2 family proteins and downstream proteases in IAV infection and replication, yet to date, no effective treatment that targets the Bcl‐2 family proteins or downstream proteases is available.

**FIGURE 4:**
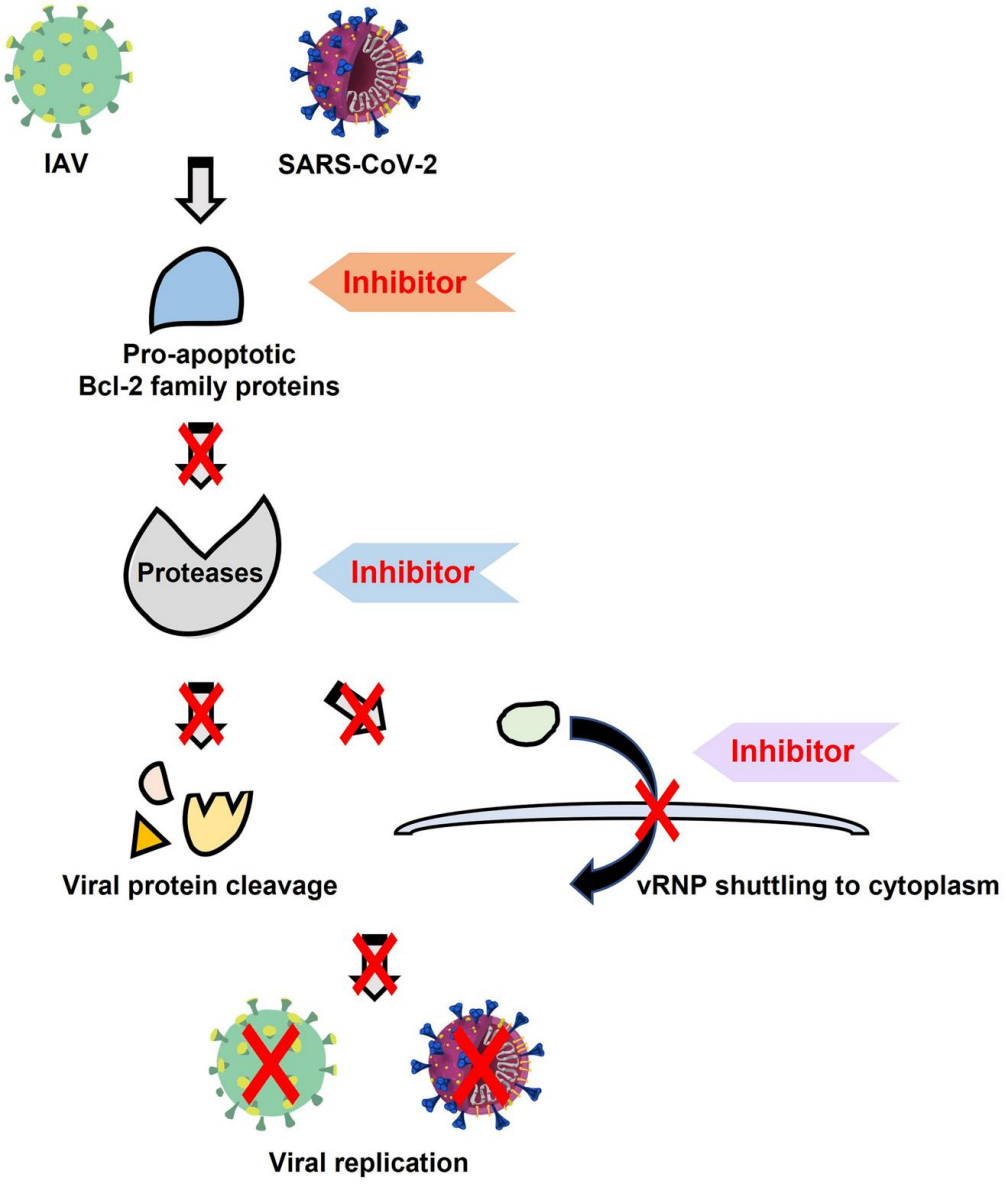
Schematic representation highlighting putative anti‐viral targets. Virus assembly and replication can be inhibited at several steps in the replication cycle of influenza A virus (IAV) and severe acute respiratory syndrome coronavirus‐2. Blocking pro‐apoptotic Bcl‐2 family proteins like Bcl‐2 interacting killer (Bik) or inhibiting proteases like caspases, cathepsins, and transmembrane proteases may impair cleavage of viral proteins mitigating viral replication and pathogenicity. Further, inhibitors may impair the nucleo‐cytoplasmic transport of viral ribonucleoprotein (vRNP) to prevent proper assembly of progeny virions resulting in reduced viral replication.

Future studies should clarify whether host cellular proteases including the ones activated by the pro‐apoptotic members of the Bcl‐2 family proteins are involved in cleavage and activation of SARS‐CoV‐2 spike proteins (Figure 4). Understanding the role of Bcl‐2 family proteins and the downstream proteases activated by these proteins will help identify inhibitors of host cellular proteases that would serve as universal treatment targets for the emerging strains of SARS‐CoV‐2 (Figure 4). Furthermore, identifying and characterising specific protease cleavage motifs of viral proteins and their specific role in IAV and SARS‐CoV‐2 replication and pathogenesis may provide possible treatment targets for infections caused by these infectious agents.

## AUTHOR CONTRIBUTIONS

Sourabh Soni and Yohannes A. Mebratu researched data, wrote the manuscript, and made the figures. All authors reviewed, edited, and approved the final version of the manuscript before submission.

## CONFLICT OF INTEREST

The authors declare no potential conflict of interest.

## Data Availability

Data sharing is not applicable to this article because no new data was created or analysed in this article.
